# Microbial Heat and Organic Matter Loss in an Aerobic Corn Stover Storage Reactor: A Model Validation and Prediction Approach Using Lumped-Parameter Dynamical Formulation

**DOI:** 10.3389/fbioe.2020.00777

**Published:** 2020-07-10

**Authors:** Carlos Quiroz-Arita, J. Austin Murphy, Mitchell A. Plummer, Lynn M. Wendt, William A. Smith

**Affiliations:** ^1^Sandia National Laboratories, Livermore, CA, United States; ^2^Idaho National Laboratory, Idaho Falls, ID, United States

**Keywords:** microbial heat, organic matter loss, corn stover, bi-phasic growth, microbial respiratory activity, storage reactor, model calibration, model validation

## Abstract

Corn stover dry matter loss effects variability for biofuel conversion facility and technology sustainability. This research seeks to understand the dynamic mechanisms of the thermal system, organic matter loss, and microbial heat generation in corn stover storage operations through system dynamics, a mathematical modeling approach, and response analysis to improve the system performance. This study considers epistemic uncertainties including cardinal temperatures of microbial respiratory activity, specific degradation rate, heat evolution per unit substrate degraded, and thermal conductivity in corn stover storage reactors. These uncertainties were managed through calibration, a process of improving the agreement between the computational and benchmark experimental results by adjusting the parameters of the model. Model calibration successfully predicted the temperature of the system as quantified by the mean absolute error, 0.6°C, relative to the experimental work. The model and experimental dry matter loss after 30 days of storage were 5.1% and 4.9 ± 0.28%. The model was further validated using additional experimental results to ensure that the model accurately represented the system. Model validation obtained a temperature mean absolute relative error of 0.9 ± 0.3°C and dry matter loss relative error of 3.1 ± 1.5%. This study presents a robust prediction of corn stover storage temperature and demonstrates that an understanding of carbon sources, microbial communities, and lag-phase evolution in bi-phasic growth are essential for the prediction of organic matter preservation in corn stover storage systems under environment’s variation.

## Introduction

Corn stover has long been recognized as a bioresource to reduce the United States’ (U.S.) dependence on foreign oil (Graham et al., 2007) and the primary feedstock for ethanol and other potential biofuels such as butanol (Qureshi et al., 2010; Green, 2011). One of the significant challenges of corn stover-derived biofuel is the variability of the feedstock, particularly in the carbohydrate content of biomass, with consequences in the biofuel yields and economics (Kenney et al., 2013). For instance, moisture content beyond 25% can contribute to dry matter losses equal to or greater than 20% due to microbial degradation of carbohydrates in storage (Kenney et al., 2013; Wendt et al., 2018). Moisture contents from 15–20%, on the other hand, have lower dry matter losses effects in biomass storage and reduce safety risks such as self-ignition (Rentizelas et al., 2009). This feedstock variability of corn stover has been demonstrated to be highly sensitive in metrics of sustainability such as life-cycle net energies, carbon dioxide emissions, and the cost of biofuels (Kim and Dale, 2005; Spatari et al., 2005; Baral et al., 2017, 2018). The effects of environmental factors in the corn stover properties such as moisture, temperature, and dry matter loss have been researched in field and laboratory studies (Wendt et al., 2014, 2018; Essien and Richard, 2018; Wang et al., 2019). These previous research efforts have demonstrated that the microbial heat resulting from degradation of carbohydrates plays a role in the corn stover thermal system and organic matter losses. However, the dynamic mechanisms between changes in the environment and the microbial kinetics in corn stover are not understood.

Many researchers have investigated microbial kinetics in composting processes (Rosso et al., 1993; Hamelers, 2004; Kulcu and Yaldiz, 2004; Richard and Walker, 2006; Richard et al., 2006; De Guardia et al., 2008; Lin et al., 2008). Others have studied kinetics in anaerobic digestion of corn stover and microbial heat evolution from glucose degradation in soil (Kimura and Takahashi, 1985; Li et al., 2016). Calorimetric research of soil microbes showed that changes in microbial growth, glucose depletion as an energy source, and the evolution of heat are proportional and can all be described as a sigmoidal curve characteristic of Monod equation (Monod, 1949; Kimura and Takahashi, 1985). As a result, the microbial heat evolution curve can express the maximum specific growth rate or specific degradation rate of the substrate because of microbial respiratory activity. Experimental results in soil, for instance, determined an average heat evolution of 1287 ± 52 KJ.mol glucose^–1^ (Kimura and Takahashi, 1985). External sources of temperature, oxygen, and moisture content, however, have been demonstrated to control the maximum specific growth rate of microorganisms (Hamelers, 2004). One of the most comprehensive studies is a cardinal temperature model with inflection that describes the mathematical representation of maximum specific growth rates in the optimal and suboptimal range of temperatures from various thermophilic, mesophilic, and psychrophilic strains grown in different media (Rosso et al., 1993; Richard and Walker, 2006). Based on the cardinal temperature model with inflection model, the cardinal temperatures for *Escherichia coli* are a minimum temperature of 4.9°C, an optimum temperature of 41.3°C, and a maximum temperature of 47.5°C. Likewise, multiple linear regression has been used to describe the mathematical representation of the half-saturation coefficient of oxygen as a function of temperature and moisture in composting, ranging from −0.67 to 1.74% O_2_ expressed in a volume percentage (v/v%) (Richard et al., 2006). Lastly, hydrolysis kinetic constants of corn stover (1-mm sieve material) in anaerobic digestion is reported at values from 0.04 to 0.17 d^–1^. The cardinal temperatures of microbial growth, moisture in suboptimal conditions, and hydrolysis and heat evolution per unit substrate kinetics have not been researched in aerobic corn stover storage environments.

State-of-the-art kinetic models of composting are mostly inductive, governed by a data-oriented approach, including first-order kinetic reactions and multiplicative environmental factors that change growth and microbial respiratory activity using composting rates (Hamelers, 2004). Heat transfer and water vapor transfer models used to predict temperature and moisture in biomass, on the other hand, are deductive or mechanistic, relying not only on data but also on the laws of physics (Hamelers, 2004; Bedane et al., 2011, 2016). These existing heat transfer and water vapor transfer models have successfully represented the physics in biomass, ignoring the connections such as the dynamic response of input heat in the growth of microorganisms. For instance, a two-dimensional model based on Fick’s diffusion equation, and the governing heat balance equation have been demonstrated to predict heat and moisture in woody biomass (Bedane et al., 2011). Furthermore, water vapor transport has been effectively predicted in a model with pore and surface diffusion as a lumped parameter at a variety of relative humidity percentages, from 10 to 90% (Bedane et al., 2016). Experiments at laboratory and field scales have illustrated the heat in corn stover storage systems’ biological and physical processes, including microbial heat, conductive, convective, and radiative heat transfer (Wendt et al., 2014, 2018). The individual heat evolution processes, both microbial and physical, and coupling mechanisms of heat in the thermal system of corn stover, are still unknown.

This research seeks to understand the dynamic mechanisms of the thermal system, organic matter loss, and microbial heat generation in corn stover storage through system dynamics, a mathematical modeling approach of systems, and response analysis to improve the system performance (Ogata, 1998). Aleatory and epistemic uncertainties must be considered and differentiated in the construction of the mathematical model (Oberkampf et al., 2004a). This study finds epistemic uncertainties, including the specific degradation rate, cardinal temperatures of microbial growth, thermal conductivity, and heat evolution per unit substrate degraded. We deal with these uncertainties through calibration, a process of improving the agreement between the computational and benchmark experimental results by adjusting the parameters of the model (Trucano et al., 2006). To assess that the model accurately represents the system, we measure the agreement between computational and a variety of experimental results through validation (Oberkampf and Barone, 2006). This study systematically assesses the predictive capability of a system dynamic corn stover storage reactor through model calibration and validation.

## Experimental and Computational Methodologies

**FIGURE 1:**
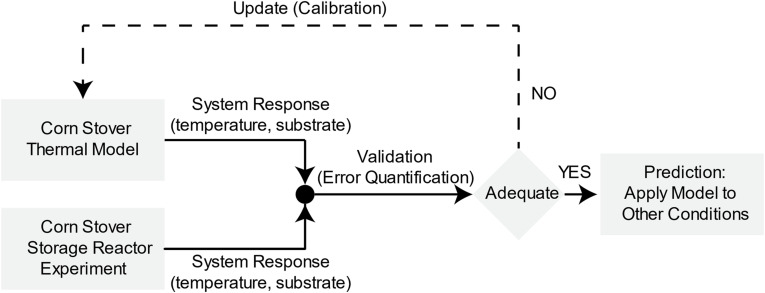
Validation, calibration, and prediction of corn stover storage reactor dynamic thermal system. Adapted from Oberkampf and Barone (2006).

To understand the dynamic mechanisms of microbial heat in aerobic corn stover storage, we must evaluate the predictive capability of the dynamic lumped thermal system following a systematic validation and calibration of the computational model with the experimental data from the corn stover storage reactors. Figure 1 illustrates the validation, calibration, and prediction approach (Oberkampf and Barone, 2006) applied to the corn stover thermal model. In this approach, first, we obtain the input quantities to develop the computational model from the experimental work in the corn storage reactors. Second, we compare the validation metric, which is system temperature and substrate, of the computational and experimental results to measure the accuracy of the model. Third, we establish an engineering decision based on the expected accuracy of the model, where a feedback loop is taken for additional calibration to reduce the error of the model relative to the experimental data. Lastly, we evaluate the predictive capability of the model with a blind computational prediction of additional corn stover storage reactor operating conditions. For an extensive study of validation, calibration, and prediction approach, see references: (Oberkampf et al., 2004a; Oberkampf and Barone, 2006). The next sections describe the methodologies of the experimental work in the corn storage reactors, computational dynamic lumped thermal system, and the validation, calibration, and prediction process.

### Corn Stover Storage Reactor Experiments

**FIGURE 2:**
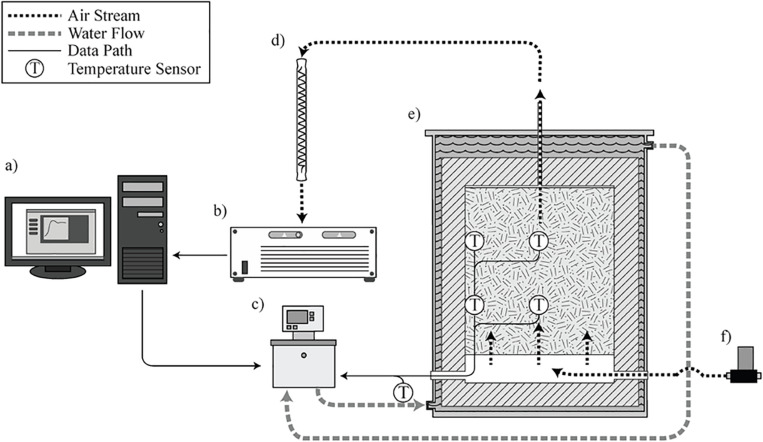
Operational illustration of the laboratory reactor system. **(a)** LabView control interface and data logging. **(b)** Gas chromatograph. **(c)** Heated water circulator. **(d)** Vapor condenser. **(e)** Reactor loaded with biomass in operation. **(f)** Mass flow controlled gas supply (Wendt et al., 2014; Bonner et al., 2015).

The laboratory-scale corn stover storage reactors studied in this research are located at Idaho National Laboratory, loaded with corn stover harvested in Hardin County, Iowa, in October 2018. The storage reactors consist of four replicates, and each reactor has a total volume of 100-L and a 76-L working volume. A complete description of the design and operation of the storage reactors can be found in: (Wendt et al., 2014; Bonner et al., 2015) and is illustrated in Figure 2.

The loaded biomass was compressed at 3.9 kPa at five 300 s intervals. Moisture content was determined by collecting five representative samples and drying at 105°C for 24 h in a Shel Lab forced air oven (Sheldon Manufacturing, Cornelius, OR, United States). Additionally, water exiting the reactors was collected and measured using a condensing column cooled with a solution of water and propylene glycol. To allow biomass drying during storage, we controlled the airflow by mass flow controllers (Brooks Instruments, Hatfield, PA, United States). The airflow rates in reactors 1 and 2 were 0.25 standard liters per minute (slpm) and 1.0 slpm in reactors 3 and 4. Airflow rates of 0.25 and 1.0 slpm were selected because they demonstrated significant differences in the microbial activity in corn stover storage systems (Wendt et al., 2014). Corn stover biomass was stored for 34 days in reactors 1 and 2 and 11 days in reactors 3 and 4.

Each reactor contained four resistance temperature detectors (RTDs) and 15 K-type thermocouple wires (Omega Engineering, Norwalk, CT, United States) placed throughout the biomass to measure temperature. Circulating water surrounded each reactor jacked set to offset the internal temperature by −0.5°C, controlled through a feedback loop between a Labview (National Instruments, Austin, TX, United States) control interface and the RTDs. Corn stover and water jacket temperatures data were collected and exported to a text file every minute from the RTDs and every 5 minutes from the thermocouples.

Gas chromatography was used to measure the concentration of O_2_, N_2_, and CO_2_ in the reactors’ off-gas with an Agilent 490 Micro GC (Santa Clara, CA, United States). Gas samples were initially collected each hour along with a sample of ambient air, and the data were exported to a spreadsheet, where they could be analyzed daily. As the biomass degradation rate decreased, reaching a quasi-steady CO_2_ production, sample frequency was decreased from one to 6 h. We assumed glucose oxidation is a suitable representation in these experiments, calculated from the CO_2_ data and the empirical formula (Porges et al., 1956) to estimate dry matter loss as follows:

$$C_{6}\,H_{12}\,O_{6}+6\,O_{2}\to 6\,C\,O_{2}+6\,H_{2}\,O+\mathrm{Heat}$$

$${\mathrm{CH}}_{2}\,O+O_{2}\to {\mathrm{CO}}_{2}+H_{2}\,O+\mathrm{Heat}$$

The corn stover temperatures measured in storage reactors and substrate degradation calculated from CO_2_ data were used for validation and calibration of the system dynamic model. For modeling purposes, the corn stover temperature, ambient temperature, loading dry matter mass, airflow rates, and moisture uncertainties are small and are treated as deterministic. The initial conditions of these model inputs are summarized in Table 1.

**TABLE 1:** Model inputs for dynamical formulation.

Parameter	Reactor 1	Reactor 2	Reactor 3	Reactor 4
Initial corn stover temperature (°C)	10.6	12.9	13.8	13.5
Ambient temperature (°C)	24.2	24.3	24.0	23.5
Loading dry matter (g)	6456	7080	7071	7290
Air flow rate (cm^3^.min^–1^)	365	347	1265	1209
Initial moisture content (%)	30.86	29.86	28.99	29.15
Final moisture content (%)	15.46	16.93	18.01	18.38
Storage time (days)	34.7	34.1	10.7	10.7

### System Dynamics Formulation

The system temperature of the corn stover storage reactor is an essential metric to the model because of its effects on the respiration of the microorganisms and in the organic matter degradation (Kimura and Takahashi, 1985; Rosso et al., 1993; Richard and Walker, 2006; Richard et al., 2006). To understand the thermal parameters that influence thermal conditions and, therefore, microbial respiratory activity, we developed a lumped thermal system model (Palm, 1983; Ogata, 1998; Incropera et al., 2007; Quiroz-Arita et al., 2020). The model of the corn stover storage reactor considers microbial heat evolution, conductive heat transfer, convective heat transfer, evaporation, and the bulk thermal capacitance of the corn stover biomass. An energy balance was carried out using a single thermal node, assuming a thermally homogeneous reactor, and the resulting ordinary differential equation was solved numerically. This dynamic thermal model is described in the following sections.

#### Microbial Heat Evolution

Heat evolution is associated with an increase in biomass growth and substrate depletion (Kimura and Takahashi, 1985). As described in section “Corn Stover Storage Reactor Experiments,” we calculated substrate degradation from the experimentally measured CO_2_ and the empirical formula of glucose used for validation and calibration of the system dynamics model. Substrate degradation modeling has been proposed as a first-order differential equation for composting processes, including multiplicative environmental factors that change the biological response (Hamelers, 2004):

(1) $$\frac{d\,S}{d\,t}=-k_{s}\cdot f\,\left(T\right)\cdot f\,\left(M\right)\cdot \left(S_{0}-S\right)$$

Where *S* is the substrate, *S*_*0*_ the initial substrate conditions, *k*_*s*_ the substrate decay rate, *f*(*T*) the temperature factor, and *f*(*M*) the moisture factor. *f*(*T*) was computed from Eq. (2) (Rosso et al., 1993; Richard and Walker, 2006), describing the substrate decay rates in the optimal (T_opt_) and suboptimal (T_max_, T_min_) range of temperatures (T) of the growth phases in the stored corn stover. T_opt_, T_max_, and T_min_ were treated as epistemic uncertainties, as described in section “Validation, Calibration, and Prediction.” *f*(*M*) was computed from the Monod Eq. (3), assuming a linear drying rate of corn stover moisture content (M) presented in Table 1 during storage. The 25% value in Eq. (3) corresponds to M at half the maximum specific growth rate, determined from dry matter loss experiments conducted at 20, 25, 30, 36, and 50%.

(2) $$f\,\left(T\right)=\frac{\left(\left(\text{T}-{\text{T}}_{\text{max}}\right)\cdot {\left(\text{T}-{\text{T}}_{\text{min}}\right)}^{2}\right)}{\begin{array}{c} (\left({\text{T}}_{\text{opt}}-{\text{T}}_{\text{min}}\right)\cdot (\left({\text{T}}_{\text{opt}}-{\text{T}}_{\text{min}}\right)\cdot \left(\text{T}-{\text{T}}_{\text{opt}}\right)\\ -\left({\text{T}}_{\text{opt}}-{\text{T}}_{\text{max}}\right)\cdot \left({\text{T}}_{\text{opt}}+{\text{T}}_{\text{min}}-2*\text{T}\right))) \end{array}}$$

(3) $$f\,\left(M\right)=\frac{\text{M}}{\left(0.25+\text{M}\right)}$$

Substrate degradation, microbial growth, and heat can be described as a sigmoidal curve characteristic of Monod equation (Monod, 1949; Kimura and Takahashi, 1985). Therefore, CO_2_ and microbial heat are proportional to the substrate degraded in the corn stover storage reactor. Anaerobic digestion modeling strategies have described hydrolysis and biogas as a first-order differential equation, including a conversion coefficient from the substrate to product (Vavilin et al., 2008; Quiroz-Arita et al., 2019). *CO*_*2*_ and microbial heat (*Q*_*m*_), therefore, were computed from Eqs. (4) and (5). The conversion coefficient (*y*_*CO2*_) from the substrate to *CO*_*2*_ is 1.44 g *CO*_2_/*S*, calculated from the experiments described in section “Corn Stover Storage Reactor Experiments.” The conversion coefficient from the substrate to microbial heat (*y*_*m*_), was treated as an epistemic uncertainty described in section “Validation, Calibration, and Prediction.”

(4) $$\frac{d\,C\,O_{2}}{d\,t}=y_{C\,O\,2}\cdot S$$

(5) $$\frac{d\,Q_{m}}{d\,t}=y_{m}\cdot S$$

#### Conductive Heat Transfer

Thermal conductivity governs the rate of heat dissipation in the corn stover storage (Karki et al., 2015). Heat transport by conduction was experimentally performed with a feedback loop through a water jacket in the corn stover storage reactor, as described in section “Corn Stover Storage Reactor Experiments” Conductive heat transfer (*Q*_*k*_) is modeled as a function of the thermal conductivity (*K*), the characteristic length (*L*), the heat flux area (A), and the net temperature difference between the corn stover and water jacket (*T*_2_−*T*_1_) following Eq. (6) (Incropera et al., 2007). The spatially distributed reactor’s temperatures obtained from the 15 K-type thermocouple wires (section “Corn Stover Storage Reactor Experiments”) demonstrate that the heat is diffusing faster near the top flange of the reactor, suggesting heat losses through the stainless-steel parts of the reactor as illustrated in Supplementary Material. *K* and A, therefore, are treated as epistemic sources of uncertainty, as explained in section “Validation, Calibration, and Prediction.” The value of *L* is 0.08 m. The water jacket temperature was obtained from the experimental work as described in section “Corn Stover Storage Reactor Experiments.” and the corn stover temperature is numerically solved, as described in section “Thermal Capacitance.”

(6) $$Q_{k}=-\frac{K}{L}\cdot \text{A}\cdot \left(T_{2}-T_{1}\right)$$

#### Convective Heat Transfer

Heat is also transported from the corn stover to the local atmosphere through convective heat transfer (Palm, 1983; Bergman et al., 2011). Convective heat transfer (*Q*_*h*_) is modeled as a function of the net temperature difference between the corn stover and the ambient temperature (*T*_2_−*T*_1_) and a heat transfer coefficient (*h*_*i*_) (Bergman et al., 2011):

(7) $$Q_{h}=-h_{i}\cdot \text{A}\cdot \left(T_{2}-T_{1}\right)$$

*h*_*i*_ (8) is estimated from the Nusselt number (*N*_*ux*_) (9), the air thermal conductivity (*k*), and *L*. The Nusselt number is a function of the Reynolds number (*Re*) and the Prandtl Number (*Pr*) (10). The Prandtl number is a function of kinematic viscosity (ν), thermal diffusivity (α) (11), thermal conductivity (*k*), fluid density (ρ), and fluid specific heat (*C*_*p*_). The Reynolds number (12) is a function of the fluid velocity (*u*), *L*, and the kinematic viscosity (ν) (Bergman et al., 2011).

(8) $$h_{i}=\frac{N_{u\,x}\cdot k}{L}$$

(9) $$N_{u\,x}=0.0296\cdot R\,e^{4/5}\cdot P\,r^{1/3}$$

(10) $$P_{r}=\frac{\nu }{\alpha }$$

(11) $$\alpha =\frac{k}{\rho \cdot C_{p}}$$

(12) $$R\,e=\frac{u\cdot L}{\nu }$$

#### Evaporation Heat Loss

Thermal energy can be lost from the system through evaporation (Incropera et al., 2007). For the case of the corn stover storage reactor, evaporation losses were measured daily, and the rate (*E*) was computed as the derivative of the condensate volume ($\frac{\Delta \,V}{\Delta \,t}$) as described in section “Corn Stover Storage Reactor Experiments.” The specific enthalpy (*h*) due to evaporation was used in the heat balance, 2257 kJ kg^–1^ evaporated water, to compute the thermal energy loss:

(13) $$E=-h\cdot \frac{\Delta \,V}{\Delta \,t}$$

#### Thermal Capacitance

The thermal capacitance (*C*_*th*_) of the corn stover biomass is defined as the capacity of the system to store thermal energy (Palm, 1983; Ogata, 1998). This characteristic is a function of thermal properties of the system including density (ρ), volume (*V*), and the specific heat (*c*(*wet*)):

(14) $$C_{t\,h}=\rho \cdot V\cdot c\,\left(w\,e\,t\right)$$

Corn stover density at sieve materials sizes of 2 mm, 4 mm, and 8 mm are reported at 942, 954, and 832 kg.m^–3^, respectively (Karki et al., 2015). The uncertainty of density is assumed negligible in the model. The volume of the corn stover storage reactor is 0.074 m^3^. Previous authors have demonstrated that the specific heat of woody biomass depends on temperature (*T*) and *M* (Ragland et al., 1991). This dependence has not been researched for corn stover. Therefore, we used the relationship for dry wood biomass [*c*(*dry*) = *KJkg*^−1^*K*^−1^] as given by Eq. (15) (TenWolde et al., 1988; Ragland et al., 1991). A correction factor term for the specific heat of wet wood biomass [*c*(*wet*) = *KJ*.*kg*^−1^.*K*^−1^] is recommended to account for the energy absorbed by the wood-water bonds as given by Eq. (16) (TenWolde et al., 1988; Ragland et al., 1991).

(15) c⁢(d⁢r⁢y)=0.1031+0.00386⋅T

(16) c(wet)=[c(dry)+4.19⋅M]/(1+M)+(0.02355⋅T−1.32⋅M−6.191)⋅M

Lastly, the thermal capacitance of stainless steel was considered in the total thermal capacitance by assuming a stainless-steel density of 7750 kg.m^–3^ and a specific heat of 480 J kg^–1^ K^–1^. The stainless-steel volume (V_ss_) was treated as an epistemic uncertainty.

#### Energy Balance and Dynamic Thermal Simulation

The heat balance (*q*_*th*_) was computed by considering *Q*_*m*_, *Q*_*k*_, *Q*_*h*_, and E following Eq. (17). The time history of the corn stover storage temperature (18) is numerically calculated using the Dormand–Prince (RKDP) method in Matlab^®^ at a variable time step for reactors 1 through 4. The theory of the RKDP numerical analysis method can be reviewed in Prince and Dormand (1981).

(17) $$q_{t\,h}=Q_{m}+Q_{k}+Q_{h}+\text{E}$$

(18) $$\frac{d\,T}{d\,t}=\frac{1}{C_{t\,h}}\cdot q_{t\,h}$$

### Validation, Calibration, and Prediction

The predictive capability of the system dynamics model is evaluated using the dataset gathered at the corn stover storage reactor, as described in section “Corn Stover Storage Reactor Experiments.” The temperatures of the corn stover and dry matter losses were used to quantify the error between model and experiment for the system dynamics model. The error of the system dynamics model, temperature and substrate, is quantified as the difference between each experimental data point (*Y*_*i*_) and the value of the model at each time step [*f*(*x*)_*i*_] (19) (Oberkampf et al., 2004b). The dynamic thermal model error was quantified by the mean absolute relative error (20). The predicted dry matter loss relative error is quantified from Eq. (21) at quasi-steady state.

(19) $$e\,r\,r\,o\,r=f\,{\left(x\right)}_{i}-Y_{i}$$

(20) $$M\,e\,a\,n\,A\,b\,s\,o\,l\,u\,t\,e\,R\,e\,l\,a\,t\,i\,v\,e\,E\,r\,r\,o\,r=1/n\cdot \sum_{i=1}^{n}\left|f\,{\left(x\right)}_{i}-Y_{i}\right|$$

(21) R⁢e⁢l⁢a⁢t⁢i⁢v⁢e⁢e⁢r⁢r⁢o⁢r=(|Y-f⁢(X)|/Y)⋅100

The parameters of the storage reactor system are calibrated using the data from reactor 2. Calibration was performed to estimate epistemic uncertainties, including the cardinal temperatures of microbial growth, the heat evolution per unit substrate degraded, substrate decay rate, heat flux area, and stainless-steel volume. These parameters, the baseline, and bounds values are summarized in Table 2. The parameters were simultaneously calibrated by minimizing the error of the model concerning the experimental data based on a cost function (22) using the Levenberg–Marquardt algorithm to solve the non-linear least-square problem with a parameter tolerance of 1e^–6^ in Matlab. For an extensive theory of the Levenberg–Marquardt algorithm, see reference: (Moré, 1978).

**TABLE 2:** Parameters, baseline, and bounds for the system dynamics model calibration.

Parameter	Baseline	Lower bound	Upper bound
T_opt_ (°C)	41.3	35	55
T_min_ (°C)	4.9	0	20
T_max_ (°C)	65	55	75
y_m_ (J.g^–1^)	10	0	Infinite
k_d_ (s^–1^)	8.9e^–6^	0	Infinite
K (W.m^–1^.K^–1^)	9	6	14
A (m^2^)	0.6	0.29	0.975
V_ss_ (m^3^)	0.01	0	Infinite

(22) $$C\,o\,s\,t\,F\,u\,n\,c\,t\,i\,o\,n=\sum e\,r\,r\,o\,r^{2}$$

These calibrated parameters were then used for model validation using the model input data (Table 1) from reactors 1, 3, and 4 (Oberkampf et al., 2004b; Ferson et al., 2008; Roy and Oberkampf, 2011). The propagated uncertainty in the overall system includes the uncertainty in inputs from the validated system dynamics model (Roy and Oberkampf, 2011).

## Results and Discussion

The results of this research are synthesized into three components. First, we present the results of the epistemic uncertainties calibration in the microbial system dynamics model, compare the substrate degraded results gathered from the experiments in the storage reactor 2, and discuss the relevance of understanding the cardinal temperatures in the bi-phasic growth in corn stover. Second, we present the results of the epistemic uncertainties calibration in the dynamic thermal model, compare the thermal results gathered from the experiments in the storage reactor 2, and discuss the implications of the laboratory-scale model in the development of field-scale storage models. Lastly, we validate the system dynamics model to evaluate predictive capability under different operating conditions. The temperature and substrate degraded error of the model is assessed for the storage reactors 1, 3, and 4. We discuss the importance of developing a better understanding of the microbial communities and biomass characteristics in the development of system dynamics models.

### Model Calibration Estimated the Epistemic Uncertainties in Bi-Phasic Microbial Substrate Degradation and Heat Generation in Corn Stover Storage

**FIGURE 3:**
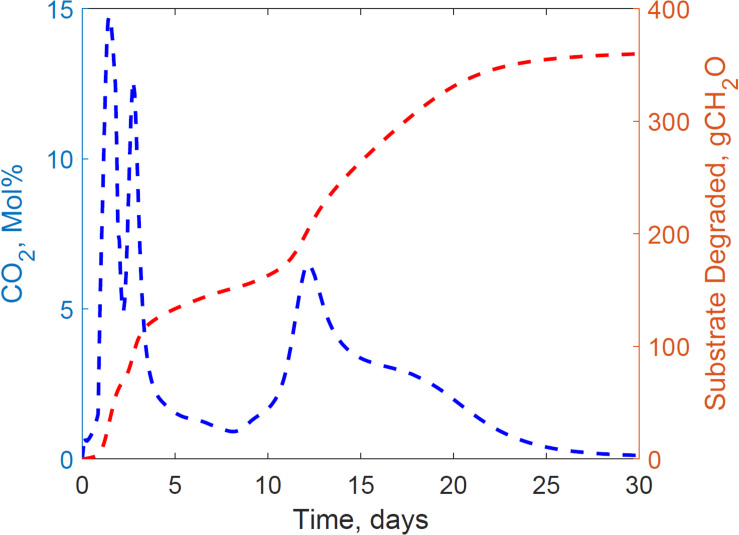
Reactor 2 experimental carbon dioxide and calculated cumulative substrate degraded calculated from empirical glucose formula demonstrating a bi-phasic growth curve in the system.

This section presents the calibration results of the substrate degradation model using the dataset gathered from reactor 2. Figure 3 illustrates the experimental CO_2_ measured through gas chromatography and the cumulative substrate degraded, calculated from the empirical glucose formula. Corn stover storage in reactor 2 exhibited a bi-phasic microbial growth, demonstrated through CO_2_ spikes at 0 and 11 days, and the bi-phasic exponential curve in the substrate degraded. Bi-phasic or diauxic growth was defined by Monod (1949) and Chu and Barnes (2016) as a bi-phasic exponential growth and intermitted lag-phase in cultivating media with two carbon sources. Monod, for instance, identified this bi-phasic growth in *E. coli* cultures with glucose and lactose media, where he observed the strain utilized lactose as a secondary carbon source after complete glucose depletion. The identification of the microbial communities, chemical characterization, and fractionation in the corn stover used in our experiments is beyond the scope of this study. While kinetics is widely researched in the literature for composting, our research is the first calibrating microbial kinetics parameters of two growth phases in corn stover storage, including the cardinal temperatures, decay rate, and heat evolution per unit glucose degraded through a system dynamics model.

**FIGURE 4:**
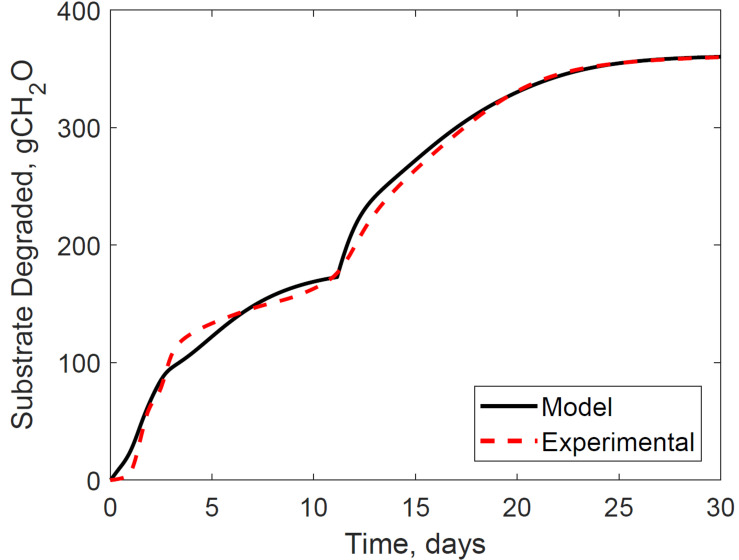
Reactor 2 substrate degraded calibration of system dynamics model. The model inputs are 347 cc.min^–1^ airflow rates, 30% initial moisture content, 17% final moisture content, 24°C ambient temperature. The initial model condition is 7080 g. The relative error is 2.0%.

**FIGURE 5:**
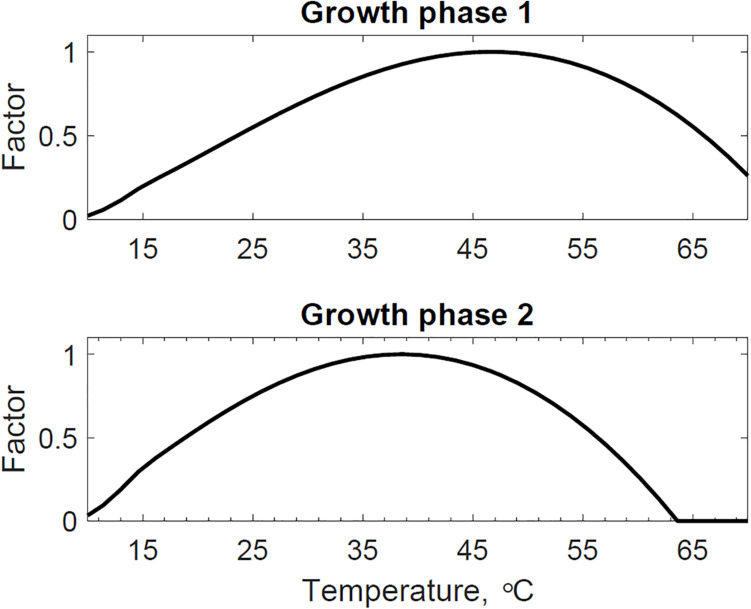
Reactor 2 calibrated cardinal temperatures for two growth phases. The first growth phase optimum, minimum, and maximum temperatures are 46.7, 4.4, and 73.6°C. The second growth phase optimum, minimum, and maximum temperatures are 38.5, 5.1, and 63.6°C.

Figure 4 presents the calibrated substrate degraded predicted by the model and compared to the experimental dataset in the storage reactor 2. Model calibration estimated 50.1% of the substrate degraded by the first growth phase, and the remaining 49.9% by the second growth phase. The calibrated lag stage of the second growth phase is 11.2 days. The model results are consistent with the bi-phasic growth curve demonstrated in the experiments, supported by Monod and Chu and Barnes (Monod, 1949; Chu and Barnes, 2016). To accomplish the agreement between model and experimental results, we assumed moisture and temperature are the environmental factors controlling the microbial respiratory activity and substrate decay rate as previously studied in composting processes by others, including Hamelers (2004); Richard and Walker (2006), and Richard et al. (2006). This modeling strategy using dimensionless environmental factors that control microbial growth in dynamic thermal and biomass systems were successfully demonstrated in predictive algal biomass models by Quiroz-Arita et al. (2020). We computed the dimensionless moisture factor from experiments at moisture content varying from 20 to 50% and Monod equation (Supplementary Figure S1), where values of one represent ideal conditions for biological activity that increase dry matter losses, and values of zero representing inhibition of biological activity, thus reducing dry matter losses. The cardinal temperatures for the dimensionless temperature factor are epistemic uncertainties in our model. Rosso L. identified a minimum temperature of 4.9°C, an optimum temperature of 41.3°C, and a maximum temperature of 47.5°C temperatures for *Escherichia coli* (Rosso et al., 1993). We used these temperatures as the baseline values for calibration, except for the maximum temperature assuming a baseline temperature of 65°C for thermophiles in an uncertain range from 55 to 75°C as supported in extensive studies of these microorganisms by Brock, T.D. (Brock, 2012). Figure 5 illustrates the calibrated cardinal temperatures for the two growth phases, one representing best microbial respiratory activity and dry matter loss conditions and zero representing inhibition.

**FIGURE 6:**
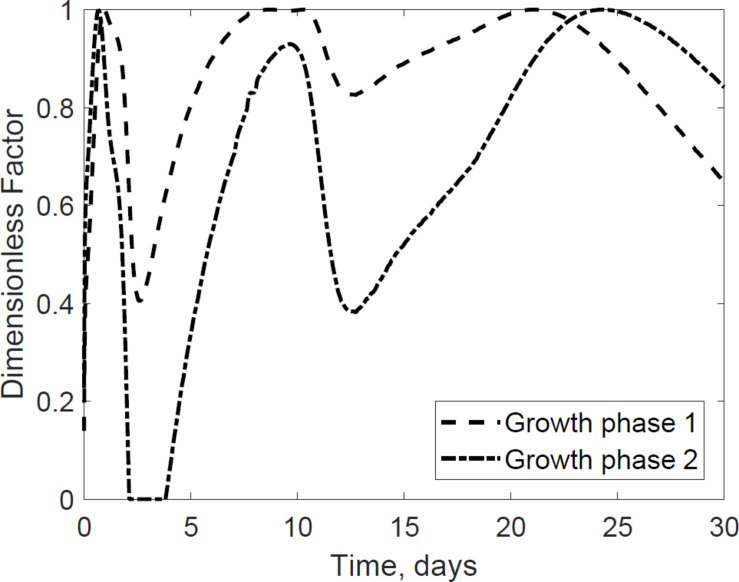
Dynamic response of microbial activity due to variations in the thermal system. The environmental temperature (dimensionless) factor represent ideal conditions for growth and organic matter loss as a value of 1. Values of zero represent inhibiting conditions that reduce microbial growth and dry matter loss. The model describes how environmental factors change through time.

**FIGURE 7:**
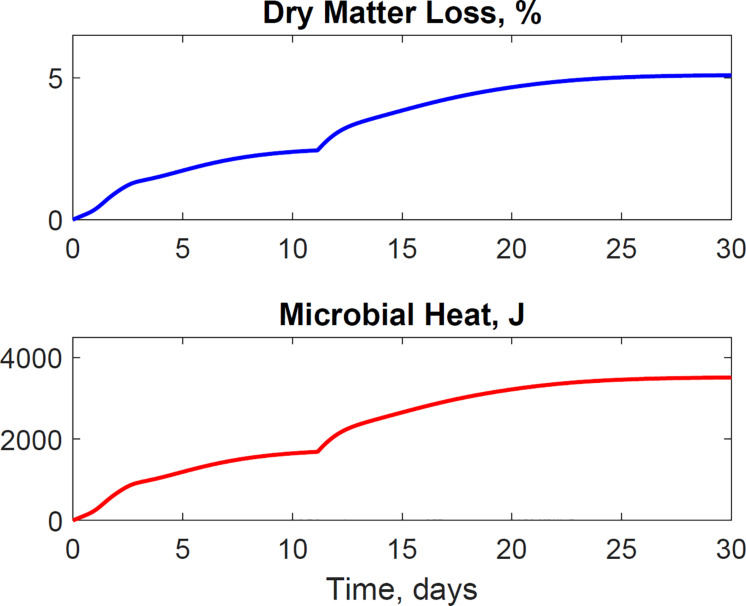
The model illustrates the dynamics of dry matter loss and microbial heat evolution. The sigmoidal microbial heat governs the temperature rise in the system. Microbial activity and heat itself are dynamically controlled for the system temperature.

The dimensionless temperature factor demonstrates that the system temperature dynamically controls microbial respiratory activity and substrate decay rate during storage. Figure 6 illustrates the dynamic response of the temperature factor in reactor 2. At time zero corn stover is at 13°C, near suboptimal temperatures for the microbial respiratory activity, 4 and 5°C in this study for the first and second growth phases, respectively, resulting in slow respiration and substrate decay rates. Microbial respiration results in substrate oxidation and heat, elevating the temperature in the system near-optimal conditions, 47 and 39°C in this study, increasing microbial respiration rates, substrate decay rates, and heat. As additional microbial heat elevates the system temperature, we approach inhibiting conditions, 74 and 64°C in this study, which results in a reduction of the system temperature. Table 3 presents the calibrated cardinal temperatures and substrate decay rates for the two growth phases in the corn stover storage reactor 2. The verification of these calibrated microbial kinetics parameters is beyond the scope of this research and can change under different environmental conditions. Figure 7 shows that the cumulative dry matter loss and microbial heat generation curves are consistent with sigmoidal growth curves, as demonstrated by Kimura and Takahashi (1985) in calorimetric studies of soil microbes. Experimental and model dry matter losses are 4.9 ± 0.28% and 5.1% as calculated from the fraction of substrate degraded of the original corn stover. Heat evolution per unit substrate degraded is an epistemic uncertainty in our model. Heat per unit substrate with other epistemic microbial kinetic parameters is calibrated to minimize the integrated error by comparison of the model to experiment substrate degraded. Our calibration process provided a heat evolution per unit substrate degraded value of 9.7 J.g^–1^, three orders of magnitude lower than values obtained by Kimura and Takahashi (1985) in soil with a glucose substrate. Environmental factors can control heat evolution, including moisture, carbon sources, and microbial and engineered feedback temperature itself. A better understanding of the initial and final organic matter characteristics and rigorous data collection of water vapor with CO_2_ can improve the validation of microbial heat in future upgrades of our model.

**TABLE 3:** Calibrated parameters for two growth phases.

Parameter	Growth phase 1	Growth phase 2
T_opt_ (°C)	46.7	38.5
T_min_ (°C)	4.4	5.1
T_max_ (°C)	73.6	63.6
k_d_ (s^–1^)	7.6e^−6^	8.3e^−6^
y_m_ (J g^–1^)	9.7	9.7
K (W m^–1^ K^–1^)	10.1	10.1
A (m^2^)	0.6	0.6
V_ss_ (m^3^)	0.01	0.01

### Model Calibration Estimated the Epistemic Uncertainties of the Corn Stover Storage Thermal System

**FIGURE 8:**
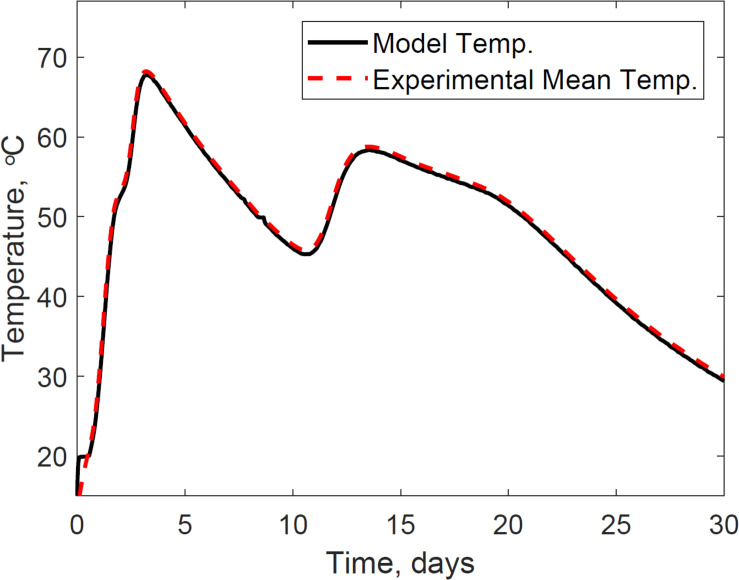
Reactor 2 temperature calibration of the system dynamics model. The model inputs are 347 cc.min^–1^ airflow rates, 30% initial moisture content, 17% final moisture content, 24°C ambient temperature. The initial model condition is 12.9°C. The mean absolute error is 0.6°C.

The thermal system consists of the microbial heat evolution results discussed in section “Model Calibration Estimated the Epistemic Uncertainties in Bi-phasic Microbial,” conductive heat transfer, convective heat transfer, evaporation heat loss, and capacitance of the system. System dynamics has been widely researched for thermal systems, our research, however, is the first applied to understand the dynamic mechanisms between the physical environment and microbial kinetics in corn stover storage reactors, calibrating epistemic parameters for this specific experiment. Figure 8 presents the calibrated system temperature predicted by the model and compared to the experimental dataset in the storage reactor 2. The system temperature demonstrates the effects of the bi-phasic microbial heat generation and substrate respiration rates discussed in section “Model Calibration Estimated the Epistemic Uncertainties in Bi-phasic Microbial.”

Epistemic microbial kinetic and physical parameters in the system are calibrated to minimize the integrated error by comparison of the model to experimental temperature. The reactor’s spatially distributed temperatures demonstrated that the heat is diffusing faster near the top flange of the reactor, suggesting heat losses through the stainless-steel parts of the reactor, convective heat transfer, and evaporation as illustrated in Supplementary Multimedia Material. Table 3 shows the calibrated thermal parameters, including thermal conductivity (*K*) and heat flux area (A) used in the conductive heat transfer, and stainless-steel volume (*V*_*ss*_) used in the thermal capacitance. Our calibration process estimated *K*, A, and *V*_*ss*_ of 10.1 W m^–1^ K^–1^, consistent with values reported in the literature for stainless steel (Incropera et al., 2007), and 0.6 m^2^ and 0.01 m^3^, physically possible for the storage reactor dimensions. Supplementary Figures S6, S7 illustrate other means of heat loss than heat diffusion, including the convective heat transfer and the evaporation heat loss. We obtained an experimental heat transfer coefficient of 2.2 W m^–2^ K^–1^, in agreement with values used in natural convection of gases reported in the literature, 2–25 W m^–2^ K^–1^ (Incropera et al., 2007). Evaporation heat loss was experimentally computed from the derivative of the condensate volume and the specific enthalpy. Lastly, Supplementary Figure S8 illustrates the specific heat of biomass. The values obtained in the model are consistent with dry wood values, 1200–1500 J kg^–1^ K^–1^ (Ragland et al., 1991), 20% moisture content wood, 1700–2300 J kg^–1^ K^–1^ (Ragland et al., 1991), and dry corn stover, 1395–1610 J kg^–1^ K^–1^ (Dupont et al., 2014). The biomass specific heat governs the capacity of corn stover to store heat in the system.

Model calibration of the thermal system model reduced the mean absolute relative error to 0.6°C, calculated from the transient error (Supplementary Figure S2). Each reactor was surrounded with a circulating water jacket set to offset the internal temperature by −0.5°C, therefore, predicted temperatures below this value could have negative implications in thermal conductivity calculations. Supplementary Figures S3, S4, for instance, illustrate time intervals during storage where corn stover temperature is above the water jacket temperature, expecting thermal diffusion from the corn stover to the surrounding water jacket. However, model corn stover temperatures in such time intervals that mispredict values below the water jacket governed the diffuse of heat from the water jacket to the corn stover. Supplementary Figure S5 illustrates these implications in the calculations of conductive heat transfer, where positive values represent the sources of errors in our model. The closed-loop feedback system controller starts at temperatures above 20°C. Therefore, heat diffusion from the water jacket to the corn stover is expected during the lag phase of microbial respiration, and heat diffusion from the corn stover to the water jacket is expected in the log phase of microbial respiration. The constraints of our model are because of the temperature control strategy in the surrounding water jacket. Under field storage conditions, however, such controlling strategies are absent, and a better prediction of conductive heat transfer between the ambient air and corn stover is expected.

### Model Validation Demonstrated the Predictive Capability of the Storage Reactor System

The performance of the system dynamics calibrated model is evaluated using a dataset not used for calibration, including reactors 1, 3, and 4. Table 1 includes the model inputs, and Table 3 presents the calibrated parameters used in model validation. We quantified the error of the system responses, temperature, and substrate degraded against the dataset gathered from experiments in reactors 1, 3, and 4. Table 4 synthesizes the system temperature and dry matter loss error quantification of the model for reactor 2, used for calibration, and reactors 1,3, and 4 used for validation. The mean absolute relative error quantifies the system temperature error, and the dry matter loss relative error quantifies the system substrate degraded error.

**TABLE 4:** Model temperature and dry matter loss error.

Parameter	Reactor 1	Reactor 2	Reactor 3	Reactor 4	Mean
Mean absolute relative error (°C)	1.2	0.6	1.1	0.8	0.9 ± 0.3
Experimental dry matter loss (%)	4.6	5.0	3.5	3.8	4.2 ± 0.7
Model dry matter loss (%)	4.7	5.1	3.4	3.6	4.2 ± 0.8
Dry matter loss relative error (%)	2.2	2.0	2.9	5.3	3.1 ± 1.5

**FIGURE 9:**
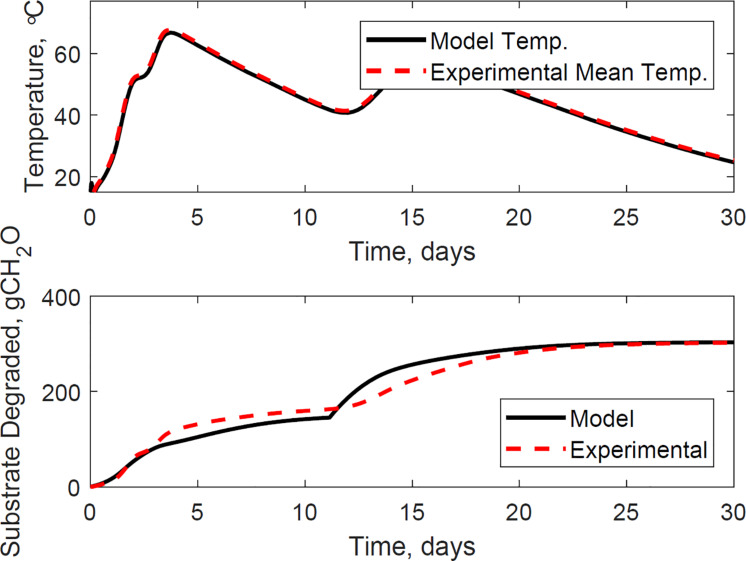
Reactor 1 temperature **(top)** and substrate degraded **(bottom)** validation of system dynamics model. The model inputs are 365 cc.min^–1^ airflow rates, 31% initial moisture content, 15% final moisture content, 24°C ambient temperature. The initial model conditions are 10.6°C and 6456 g. The temperature mean absolute error is 1.2°C, and the dry matter loss relative error is 2.2%.

**FIGURE 10:**
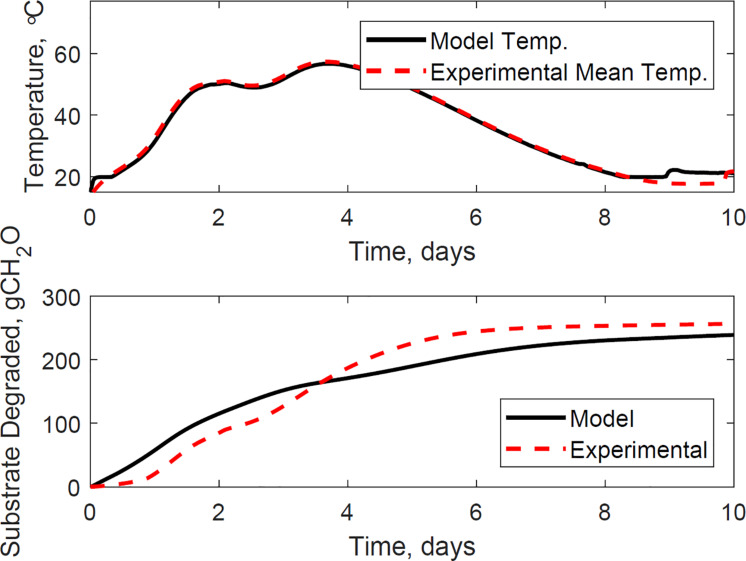
Reactor 3 temperature **(top)** and substrate degraded **(bottom)** validation of system dynamics model. The model inputs are 1265 cc.min^–1^ airflow rates, 29% initial moisture content, 18% final moisture content, 24°C ambient temperature. The initial model conditions are 13.8°C and 7071 g. The temperature mean absolute error is 1.1°C, and the dry matter loss relative error is 2.9%.

**FIGURE 11:**
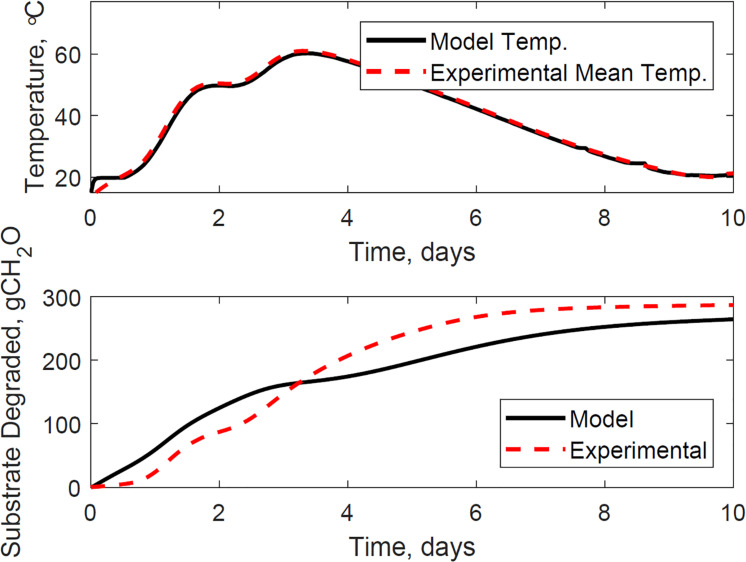
Reactor 4 temperature **(top)** and substrate degraded **(bottom)** validation of system dynamics model. The model inputs are 1209 cc.min^–1^ airflow rates, 29% initial moisture content, 18% final moisture content, 24°C ambient temperature. The initial model conditions are 13.5°C and 7290 g. The temperature mean absolute error is 0.8°C, and the dry matter loss relative error is 5.3%.

Figure 9 illustrates the evaluation of the model using the dataset of reactor 1, which was not used for calibration. The calibrated model successfully predicted the temperature and substrate degraded of the system, where the mean absolute relative error is 1.2°C, and the dry matter loss relative error is 2.2%. Reactor 1 presents the bi-phasic growth characteristics of Reactor 2. Therefore, the substrate degraded fraction for each growth phase, and the lag stage of the second growth phase estimated for reactor 2 is valid for reactor 1. This validation was expected as both reactors 1 and 2 have similar initial conditions and model inputs, and operated airflows at 356 ± 13 cm^3^.min^–1^. Figures 10, 11 illustrate the evaluation of the model using the dataset of reactors 3 and 4, with operated airflows at 1237 ± 40 cm^3^.min^–1^. Reactor 3 temperature mean absolute error is 1.1°C, and the dry matter loss relative error is 2.9%. Reactor 4 temperature mean absolute error is 0.8°C, and the dry matter loss relative error is 5.3%. The calibrated model successfully predicted the system temperature of reactors 3 and 4. However, the calibrated model has constraints to represent the bi-phasic growth in the substrate degraded, which reached a steady state in a shorter residence time than reactors 1 and 2. As a result, the substrate degraded fractions for the two growth phases, and the lag stage for the second growth phase obtained through calibration in reactor 2, are not valid under higher airflow rates used for reactors 3 and 4.

Although a robust predictive capability of the calibrated model is demonstrated for the system temperature in reactors 1, 3, and 4, a higher degree of uncertainty in the substrate degraded is observed under different environments in reactors 3 and 4. We assumed in our model two growth phases represented by two differential equations, and the calibrated kinetic parameters predicted the substrate degraded for reactors 1 and 2. For reactors 3 and 4, a single differential equation and the kinetic parameters calibrated for the second phase predicted the substrate degraded with relative errors of 2.9 and 5.3% but cannot accurately represent the bi-phasic growth observed in the experimental data. The studies of the carbon sources and microbial communities existing in the experiments are beyond the scope of this research. Additionally, a better understanding of aerobic corn stover storage systems requires a more comprehensive study of the microbial response to electron acceptor variation (O_2_) and lag-phase evolution. For instance, a recent study of Chu and Barnes (2016) demonstrated tradeoffs between adaptation and high growth rates in bi-phasic growth, with longer lag-phase in environments where switching carbon sources in less frequent and shorter lag-phase in environments where switching carbon sources is more frequent. These findings and the constraints observed in our system dynamics model highlight the need to refine the model inputs, including the existing carbon sources and microbial strains, and develop a better understanding of lag-phase adaptation in corn stover storage systems.

## Conclusion

Calibration and validation of an aerobic storage reactor system demonstrated an average predictive temperature mean absolute relative error of 0.9 ± 0.3°C and dry matter loss relative error of 3.1 ± 1.5%. The thermal and substrate degraded models were calibrated using data set from reactor 2, and the predictive capability was demonstrated using data sets from reactors 1, 3, and 4. These models show that lumped-parameters assumptions for thermal and substrate degraded in corn stover storage reactors are well-founded. The constraints of our model indicate the importance of developing a better understanding of the initial and final carbon sources, and rigorous data collection of water vapor with CO_2_ to validate microbial heat. Additionally, model development under field storage conditions is expected to contribute to a better prediction of conductive heat transfer between the ambient air and corn stover. Lastly, a comprehensive characterization of carbon sources and microbial communities, and lag-phase study in corn stover storage systems will expand the predictive capability of the model under other spectrums in the environment. This contribution will allow us to scale the model to field conditions incorporating seepage, convective heat transfer under wind and bale orientation, precipitation, evaporation, and radiation. Future model development under field conditions will contribute to engineering strategies to control microbial activity, minimize dry matter loss, reduce variability for biofuel conversion facility, and improve technology sustainability.

## Data Availability Statement

The raw data supporting the conclusions of this article will be made available by the authors, without undue reservation, to any qualified researcher.

## Author Contributions

CQ-A was the lead author, developed the system dynamics model, performed the model calibration and validation, and drafted the manuscript. JM and WS performed the storage reactors operation and data acquisition. CQ-A, JM, MP, LW, and WS performed the data analysis. LW and WS revised the manuscript. All authors contributed to the article and approved the submitted version.

## Conflict of Interest

The authors declare that the research was conducted in the absence of any commercial or financial relationships that could be construed as a potential conflict of interest.
