# Supplementary material for: Microbial Heat and Organic Matter Loss in an Aerobic Corn Stover Storage Reactor: A Model Validation and Prediction Approach Using Lumped-Parameter Dynamical Formulation
Source: Front Bioeng Biotechnol. 2020 Jul 10;8:777. doi: 10.3389/fbioe.2020.00777 (PMC7365952; doi:10.3389/fbioe.2020.00777)
Supplement: Supplementary file 2 [file Data_Sheet_1.PDF]

Supplementary material of Microbial heat and organic matter loss in an aerobic corn stover storage reactor: A model validation and prediction approach using lumped-parameter dynamical formulation

Carlos Quiroz-Arita[1*], J. Austin Murphy[2], Mitchell A. Plummer[2], Lynn M. Wendt[2], William A. Smith[2*]

[1] Sandia National Laboratories, Livermore, CA 94550, United States

[2] Idaho National Laboratory, Idaho Falls, ID, United States

CORRESPONDENCE:

Carlos Quiroz-Arita
cquiroz@sandia.gov
carlos.quiroz@fulbrightmail.org

William Smith
william.smith@inl.gov

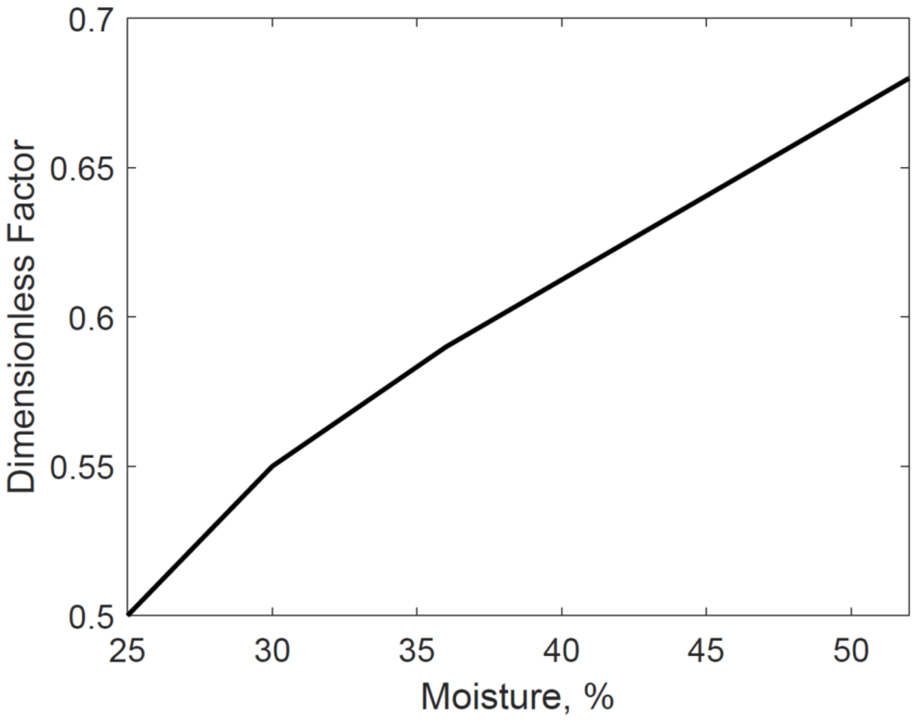

Figure S1. Reactor 2 response to microbial activity (dimensionless) due to moisture content. The moisture factor represents ideal conditions for growth and organic matter loss as a value of 1. Values of zero represent inhibiting conditions that reduce microbial growth and dry matter loss.

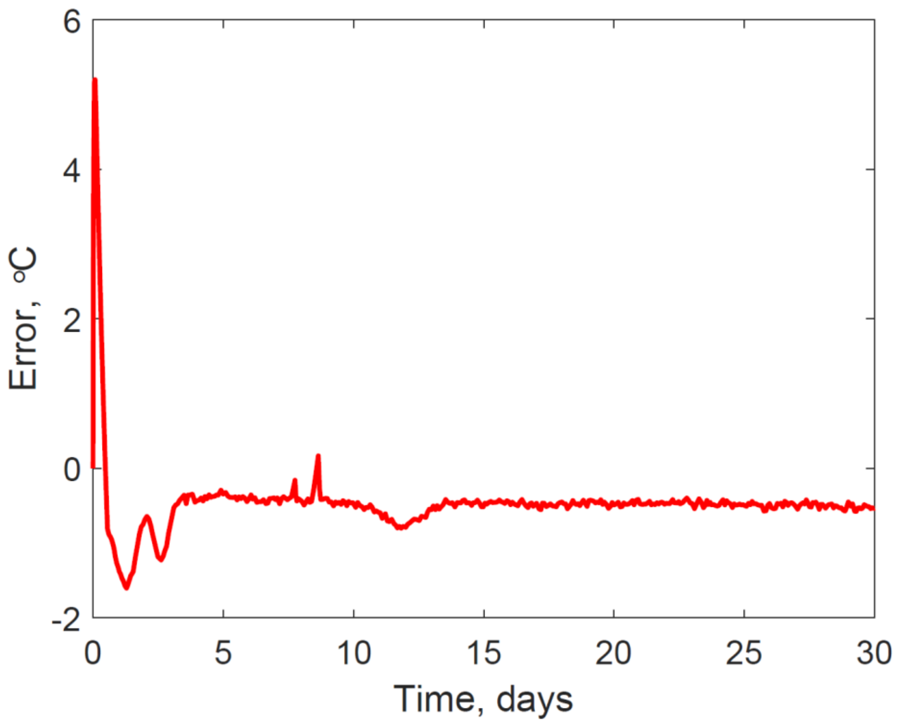

Figure S2. Reactor 2 predictive temperature error calculated from Equation (19).

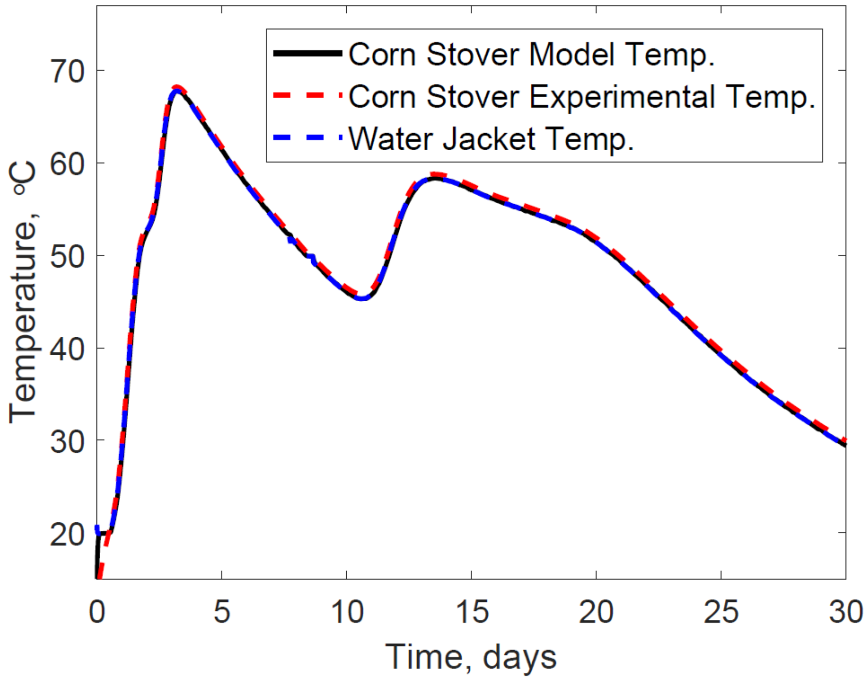

Figure S3. Reactor 2 Experimental corn stover and water jacket temperature and model temperature.

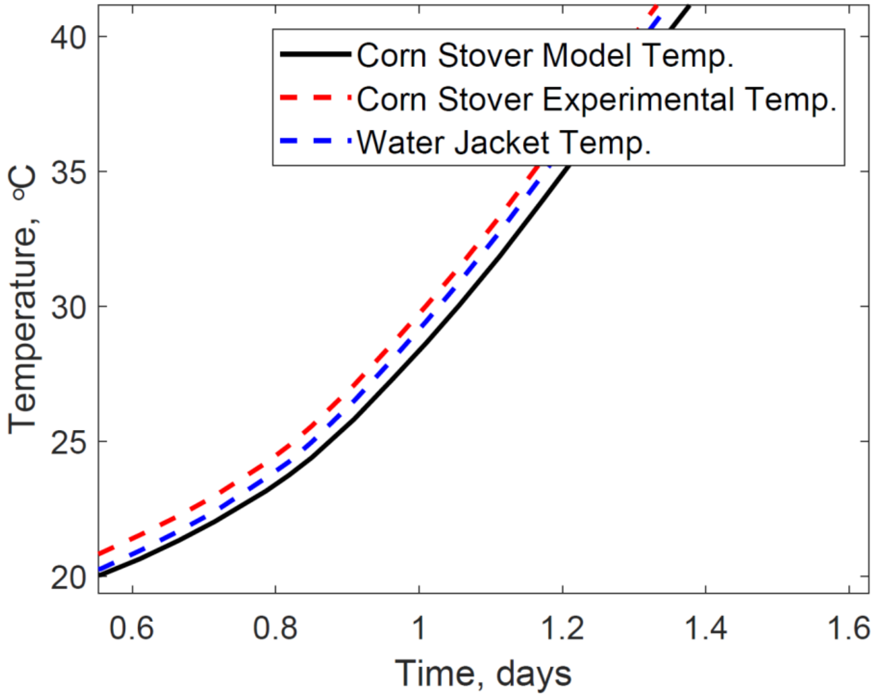

Figure S4. Reactor 2 under prediction of temperature model relative to experimental corn stover and water jacket temperatures.

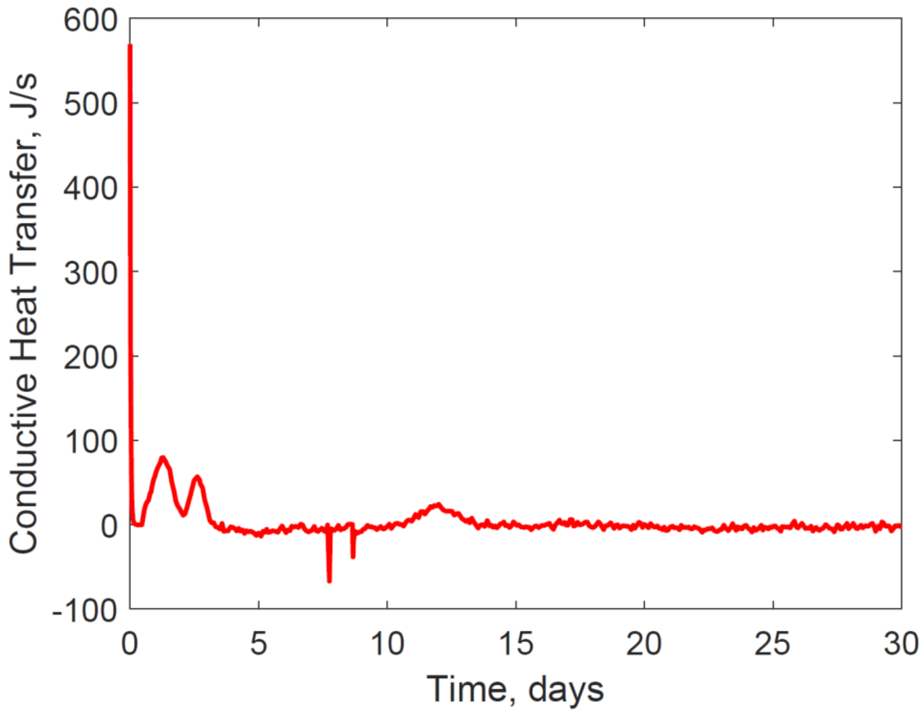

Figure S5. Reactor 2 conductive heat transfer. Positive values represent theoretical heat diffusion from the water jacket to the corn stover, and negative values represent heat diffusion from corn stover to the water jacket.

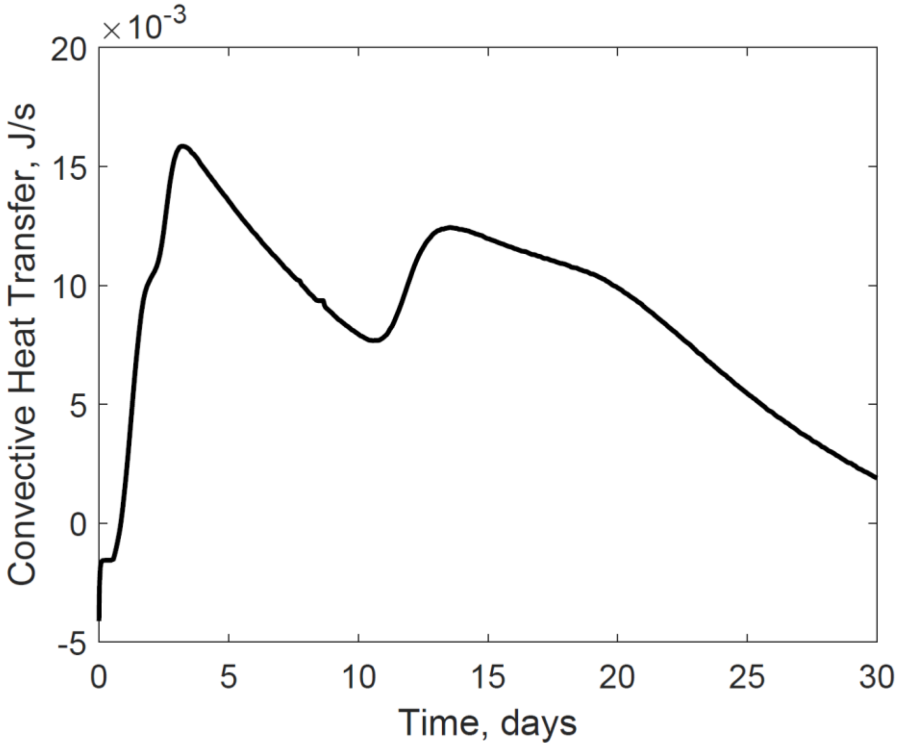

Figure S6. Reactor 2 convective heat transfer. Positive values represent heat loss from the corn stover to the ambient. Negative values represent corn stover temperatures below ambient temperatures.

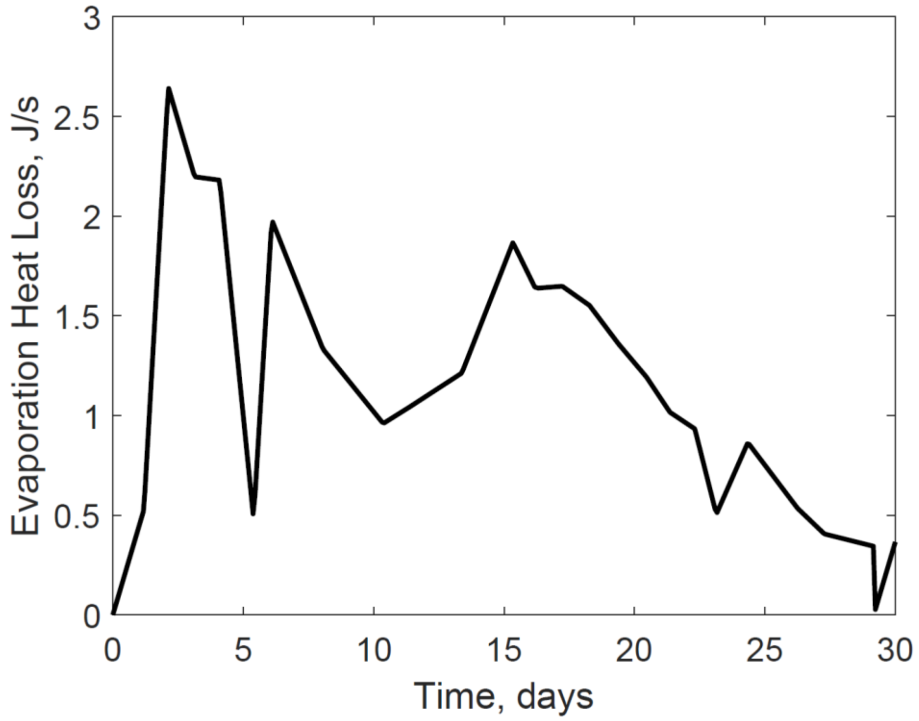

Figure S7. Reactor 2 evaporation heat loss.

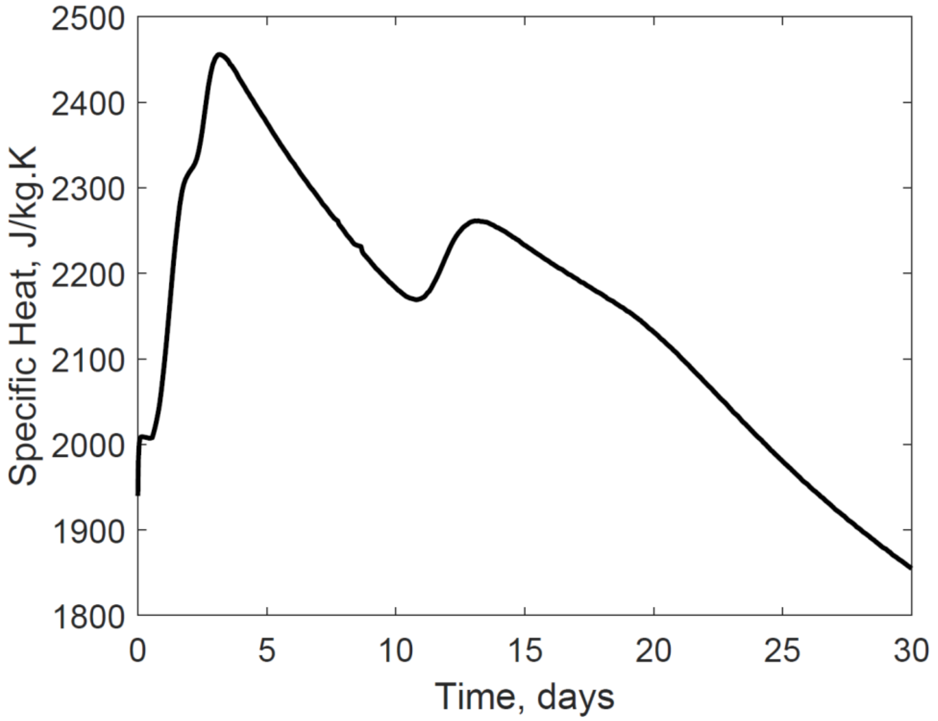

Figure S8. Reactor 2 biomass specific heat computed as a function of the system temperature and moisture.
